# A review of oral biopsies in children and adolescents: A clinicopathological study of a case series

**DOI:** 10.4317/jced.51122

**Published:** 2013-07-01

**Authors:** Edivânia B. Vale, Flávia MM. Ramos-Perez, Gabryelle LC. Rodrigues, Elaine JA. Carvalho, Jurema FL Castro, Danyel EC. Perez

**Affiliations:** 1 DDS, MSc. School of Dentistry, Department of Clinical and Preventive Dentistry, Oral Pathology Section, Federal University of Pernambuco, Recife, Brazil; 2 DDS, PhD. School of Dentistry, Department of Clinical and Preventive Dentistry, Oral Pathology Section, Federal University of Pernambuco, Recife, Brazil; 3 School of Dentistry, Department of Clinical and Preventive Dentistry, Oral Pathology Section, Federal University of Pernambuco, Recife, Brazil

## Abstract

Objective: The aim of this study was to evaluate the clinicopathological features of oral lesions in children and adolescents diagnosed in an Oral Pathology Laboratory. Study design: Between 2000 and 2010, all oral lesions diagnosed in patients younger than 18 years old, from the Oral Pathology Laboratory, Federal University of Pernambuco, Brazil, were selected for the study. The clinical data were obtained from the patient charts filed in the Laboratory. All cases were microscopically reviewed and the diagnosis classified into 10 categories. Results: From the 2395 lesions, 315 (13.1%) occurred in this age group. The lesions were more common in the female gender (59%) during the second decade of life (69%). The inflammatory/reactive lesions were the most common (64.4%), followed by the epithelial and soft tissue neoplasms (8.6%). The mucocele (33.3%) was the most common lesion, with the lip mucosa representing the most affected site (48%). In 61.5% there was concordance between clinical hypothesis of diagnosis and histopathological diagnosis. Conclusions: Inflammatory/reactive lesions were the most common biopsied lesions and the lip the most frequent site. Similar studies are important, reinforced by the low correlation between clinical diagnosis and histopathological diagnosis.

** Key words:**Adolescents, children, differential diagnosis, oral diseases, prevalence.

## Introduction

Children and adolescents may present various oral lesions, with clinical features, symptomatology, behaviour and prevalence differing from those that occur in adults (1-4). However, there are few series that relate the prevalence of oral and maxillofacial diseases in this population (1-5), with the majority of them limited to conducting epidemiological surveys about dental and periodontal diseases, such as dental caries, periodontal disease, malocclusion and dental traumas (2,3,6). In addition, some studies have been restricted to identifying and relating series of specific groups of diseases, such as odontogenic tumors, bone lesions and salivary gland diseases (1-3,7).

There is considerable variation in the prevalence of these lesions among the different regions of the world (1,5,6), as racial and environmental specificities and the lifestyle of each population may influence the prevalence of these diseases. Moreover, another important factor is the lack of standardization of age limit for considering the patient within the group of children and adolescents.

Studies on the prevalence of oral and maxillofacial lesions in children and adolescents are important for characterizing the most frequent lesions in this population. Moreover, the sites and age-ranges most affected, and the signs and symptoms of diseases differ from the oral lesions most common in adults. Thus, the aim of this study was to evaluate, retrospectively, the clinicopathological features of the oral lesions diagnosed in an oral pathology laboratory in Brazil.

## Material and Methods

Between 2000 and 2010, from 2,395 cases diagnosed in the Oral Pathology Laboratory, Federal University of Pernambuco, Recife, Brazil, 315 (13.1%) occurred in 307 patients younger than 18 years old. The clinical data, such as age, gender, time of complaint, site and size of the lesions and clinical diagnosis were obtained from the patient charts filed in the Oral Pathology Laboratory. This study was approved by the local Research Ethics Committee.

All cases were histologically reviewed by an oral pathologist in slides stained with hematoxylin and eosin. Those cases with inadequate staining due to longtime filing, new histological sections were obtained of the paraffin-embedded tissue blocks and stained with hematoxylin and eosin. Immunohistochemical reactions were performed in cases of spindle-cell mesenchymal neoplasms in order to determine the histogenesis and final diagnosis. Particularly in this series, these reactions were performed in the cases of neurilemmoma and neurofibroma.

The histopathological diagnoses were classified in 10 categories, as follow: inflammatory/reactive lesions, pigmented/melanocytic lesions, benign bone lesions, developmental lesions, autoimmune diseases, inflammatory periapical lesions, odontogenic cysts, odontogenic tumors, epithelial and soft tissue neoplasms, and normal tissue.

In the bone lesions, the final diagnosis was established after the association of the clinical, radiographic and histopathological features. In the cases with unavailable clinical and/or radiographic features, an unspecific diagnosis was established, such as odontogenic cyst and fibro-osseous lesion not otherwise specified.

## Results

There were a total of 315 lesions that occurred in 307 patients. Of these, 127 (41.4%) were in the male gender and 180 (58.6%) in the female gender (male: female ratio 1:1.4). The mean age was 12.42 years (ranging from 3 to 18 years), with 216 cases (68.57%) being presented during the second decade of life. The female gender was the most prevalent in 6 (60.0%) diagnostic categories. The frequency of lesions according to the diagnosis group is shown in  Table 1. The labial mucosa was the most affected site (151 cases – 47.93%), with reacti-ve/inflammatory lesions representing the most common diseases in this location (126 cases – 40.0%) ( Table 2).

**Table 1:**
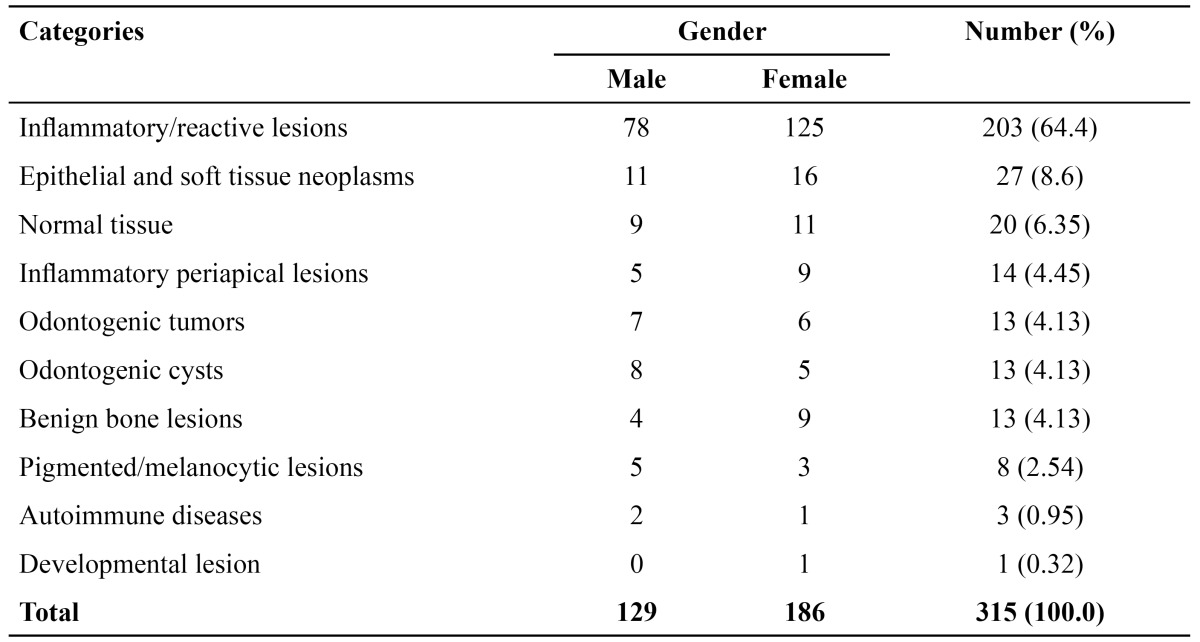
Distribution of the lesions according to diagnosis categories.

**Table 2:**
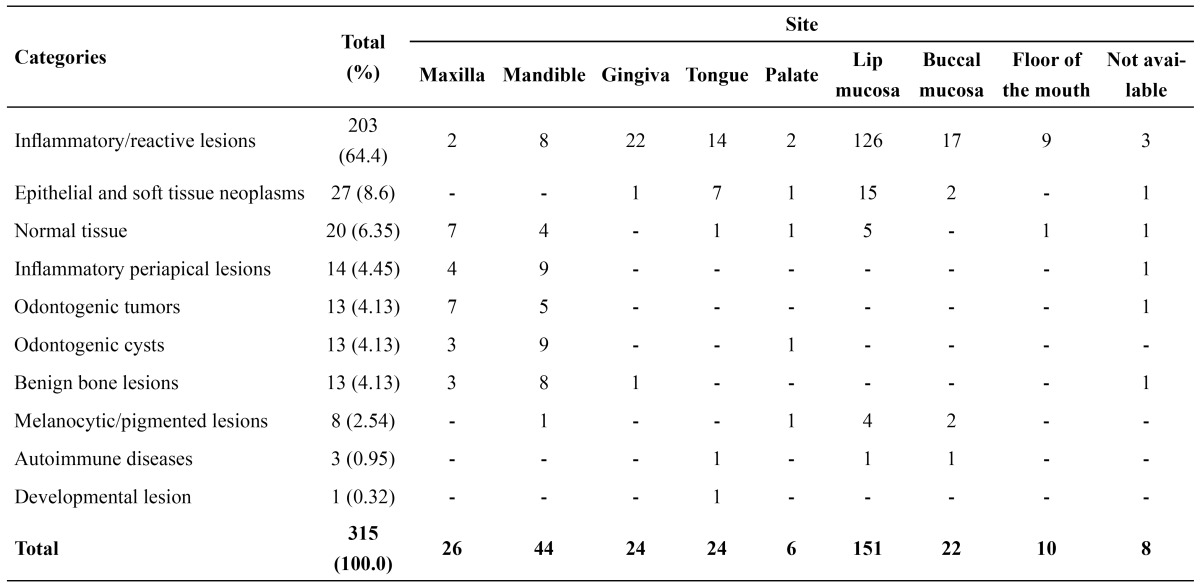
Distribution of the diagnosis categories according to the site.

Among the reactive/inflammatory lesions, mucocele (105 cases – 51.72%) was the most prevalent, followed by nonspecific chronic inflammatory reaction (22 cases – 10.83%) and fibrous hyperplasia (18 cases – 8.86%) ( Table 3). The mean age of patients with mucocele was 11.45 years (ranging from 5.0 to 18.0 years) and the majority of cases occurred in the lower lip (93 cases – 88.57%). All the cases of mucocele were the consequence of a mucous extravasation phenomenon. Microscopically, in 30 cases of mucocele (28.57%), no minor salivary glands were observed.

**Table 3:**
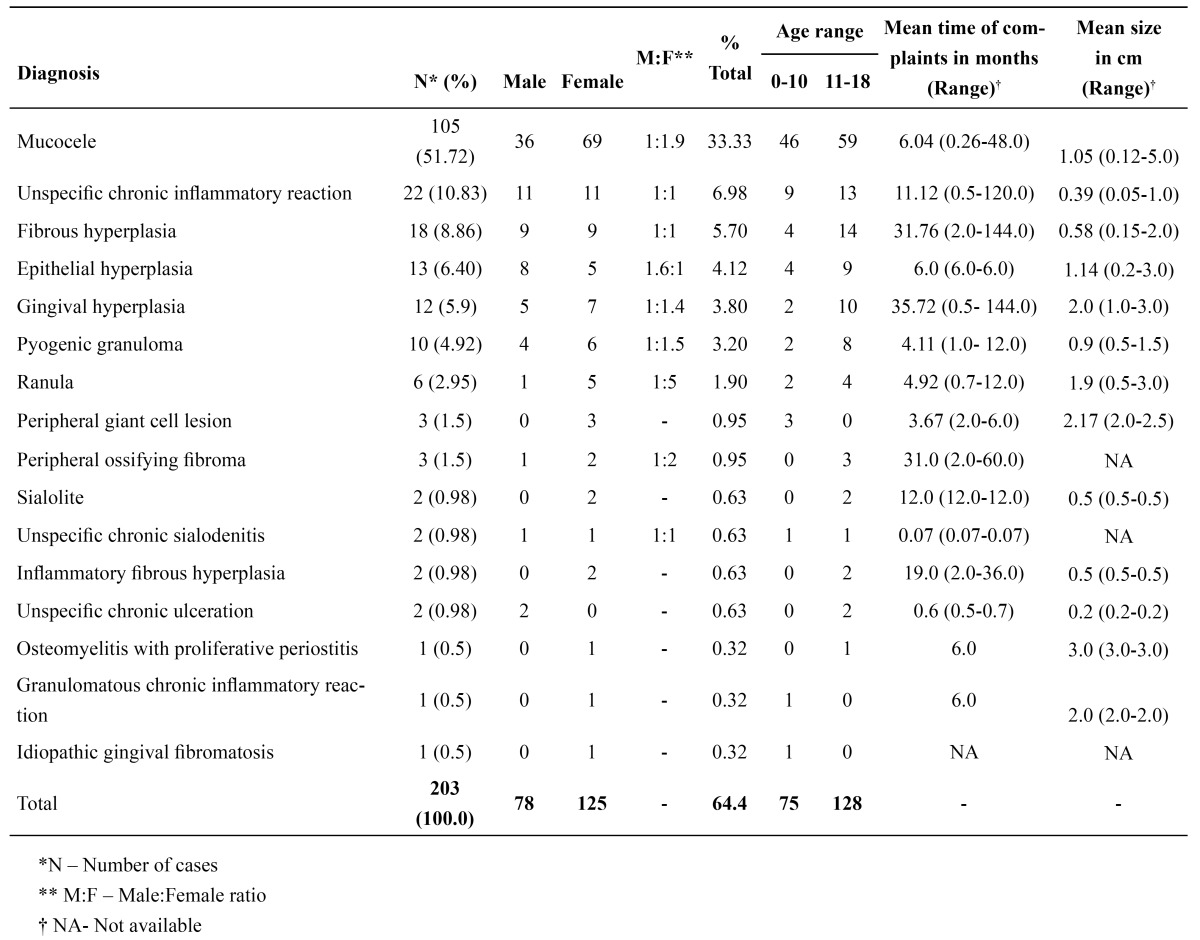
Clinical and epidemiological data of the inflammatory/reactive lesions.

Among the epithelial and soft tissue neoplasms, papilloma (12 cases – 44.4%) was the most commonly diagno-sed lesion, followed by hemangioma (6 cases – 22.2%) and lymphangioma (4 cases – 14.9%) ( Table 4). In the papillomas, the mean age of the patients was 11.75 years (ranging from 6.0 to 18.0 years) and the lower lip (7 cases – 58.4%) was the most affected site. Similarly, hemangiomas were also more frequently found in the lower lip. However, the majority of cases occurred in the first decade of life, and the mean age was 9.83 years (ranging from 5.0 to 14.0 years). Other tumors, such as neurofibromas, neurilemmoma, pleomorphic adenoma and fibrolipoma, were also diagnosed ( Table 4).

**Table 4:**
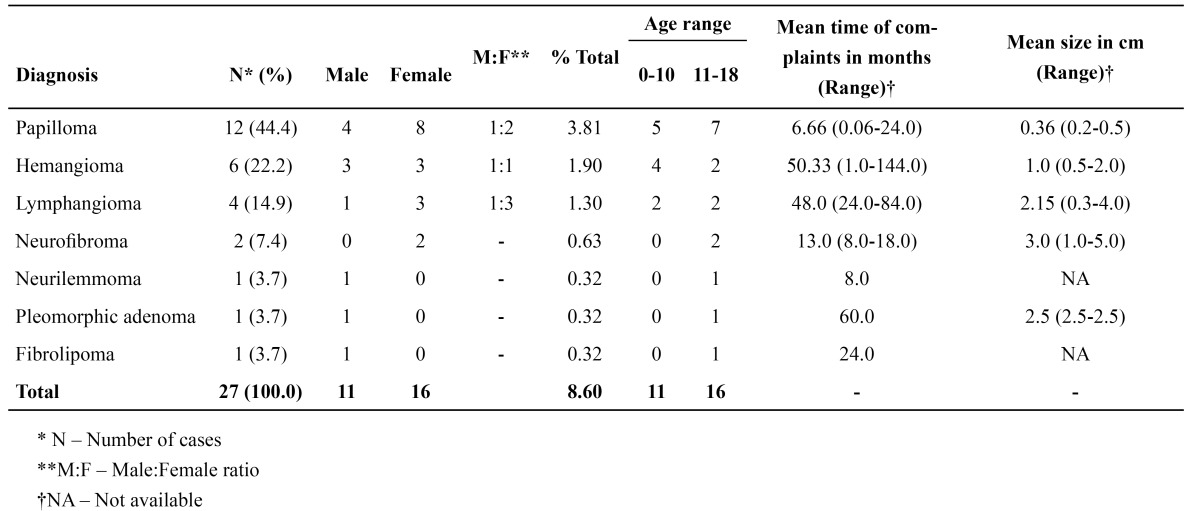
Clinical and epidemiological data of the epithelial and soft tissue neoplasms.

Periapical granuloma represented 57% (8 cases) of the inflammatory periapical lesions, followed by radicular cysts (6 cases – 43%). Periapical granuloma presented predilection for the female gender (6 cases – 75%), while radicular cyst presented no gender predilection. The age range most affected by both diagnoses was 11-18 years. The maxilla (4 cases – 50%) and mandible (4 cases – 50%) were equally affected by periapical granuloma and the mandible was affected in 83.3% (5 cases) of the radicular cysts.

Among the benign bone lesions, central giant cell lesion (6 cases – 46.15%) was the most prevalent disease, followed by ossifying fibroma (3 cases – 23.07%). Central giant cell lesion presented predilection for the female gender (83.3% – 5 cases), mean age of 14.33 years (ranging 9.0 to 18.0 years) and affected the mandible in 83.3% of the cases (5 cases). Ossifying fibroma was also more prevalent in the female gender (2 cases – 66.7%). All the individuals affected were in the age range from 11-18 years, and their mean age was 14.66 years (ranging from 12.0 to 18.0 years), with the mandible being the most affected site (2 cases – 66.7%).

Dentigerous cyst was the most prevalent odontogenic cyst (7 cases – 53.84%), most commonly affecting the female gender (4 cases – 57.14%) and the mandible (5 cases – 71.42%) of patients during the first decade of life, with a mean age of 10.85 years (ranging from 10.0 to 13.0 years). In 5 cases (38.46%), the clinical and radiographic information was not sufficient to determine the diagnosis of the lesions, these being classified as odontogenic cysts not otherwise specification. Another case (7.7%) was diagnosed as paradental cyst and occurred in the mandible of a 12-year old male patient.

Odontogenic tumors represented 4.13% (13 cases) of the cases, with the adenomatoid odontogenic tumor (3 cases – 23.07%) and compound odontoma (3 cases – 23.07%) the most frequently found diagnosis, followed by solid ameloblastoma (2 cases – 15.38%) and calcifying cystic odontogenic tumor (2 cases – 15.38%).

Pigmented/melanocytic, autoimmune lesions and those of development origin were rarely observed. Melanic pigmentation corresponded to 75% (6 cases) of the cases of pigmented lesions, while pemphigus vulgar, epidermolysis bullosa and Crohn’s disease were the autoimmune diseases diagnosed, with 1 case each. Bone choristoma was the only development lesion diagnosed, which occurred on the tongue of a 16-year-old female patient.

Pericoronal follicule and normal salivary gland were the most common diagnoses in the normal tissue category (6 cases – 30%). Normal bone tissue (5 cases) and oral mucosa (3 cases) were also identified.

Eight patients presented two simultaneous lesions, which were located either in the same anatomic site or on contralateral sides. The correlation between the clinical hypothesis of diagnosis and the histopathological diag-nosis was observed in 179 cases (61.5%), whereas 112 (38.5%) no correlation was found. In 22 cases, the spe-cimens were sent for the Oral Pathology Laboratory without a clinical diagnosis.

## Discussion

The prevalence of oral lesions in children and adolescents presents considerable variation according to studies conducted in different geographic regions, with prevalence of all oral lesions ranging between 5.5 and 24.8% (1-3,5,8,9). This disparity among the different studies may be due to the inclusion criteria. The fact that studies are not uniform with regard to criteria such as age range and diagnostic categories into which the lesions are grouped, makes it difficult to do direct comparisons (1,8). Other limitation is the lack of detailed clinical features of the lesions, particularly when the data come from an oral pathology laboratory. In the present survey it was observed that 13.1% of the oral lesions occurred in childhood and adolescence, a percentage within the range of variation found by other authors (1,3,8,10).

In the present study, the oral lesions in children and adolescents presented predilection for the female gender, differently from other authors (2,11). Nevertheless, various series observed no predilection for gender (3,5,8,9). In addition, the lesions were more prevalent during the age-range from 11-18 years, similar to that observed by the majority of the studies (1-3,5).

The reactive/inflammatory lesions were the most frequently diagnosed, similar to other series (3,9,12,13). However, similar studies conducted in an oral pathology laboratory in United Kingdom (1) and Thailand (8) indicated the dental pathologies (22.0%) and cystic lesions (35.0%) as the lesions most commonly found in children and adolescents, respectively. All these lesions are treated by dentists in their dental office. On the other hand, most of the neoplastic lesions are treated by a dentist or a physician in a Medical Hospital. For this reason, a study performed in a surgical pathology service revealed that the benign neoplasms were the most common lesions in this age group (2). These findings highlight the differences in the prevalence according to local of the study.

Mucoceles by mucous extravasation represented the most diagnosed diseases within the category of reacti-ve/inflammatory lesions, as it was also observed by Dhanuthai et al. (8). Moreover, taking into consideration the total sample, mucoceles were the most common oral lesions in children and adolescents, similar to the majority of series (3,5,7,12,14). In addition, most of these lesions were found in the lower lip, a data that reinforces the findings of previous studies (15,16). An interesting finding in this present series was the absence of minor salivary glands in almost 30% of the specimens, which indicates an inadequate treatment of the disease. It is worth pointing out that the minor salivary glands associated with the lesion should be removed to prevent recurrence of the lesion. Fibrous hyperplasia is another common oral inflammatory/reactive lesion in this age-range (17).

The prevalence of neoplasms varies significantly according to the origin of the study. Surveys performed in oral pathology laboratories indicate lower prevalence of neoplasms, when compared with those conducted in hospitals, which show greater prevalence of both benign and malignant tumors (1,2,8,13,18). In this study, the epithelial and soft tissue neoplasms comprised the second most prevalent diagnostic category, with papilloma representing the most common neoplasm, followed by hemangioma. In contrast, Wang et al. (3) observed a prevalence of only 3.4% papillomas among the benign oral neoplasias. Jones and Franklin (1) observed that hemangioma corresponded to over 40% of the lesions grouped in the category of conjunctive tissue diseases, whereas Lawoyin (2) observed that hemangiomas corresponded to only 1.1% of benign soft tissue tumors. As hemangiomas cannot always be biopsied, their prevalence is probably higher than the number of cases reported in similar studies (5). Although salivary gland tumors are rare in children and adolescents, special attention should be paid to submucous nodules, particularly those located in the palate, since the prevalence of benign and malignant tumors of the salivary gland is similar in this age group (18). In this study, only one case of pleomorphic adenoma was diagnosed.

Among the cystic odontogenic lesions, the dentigerous cyst was the most common, affecting mainly patients of the female gender of up to 10 years of age. This result is similar to that of other studies, with this lesion corres-ponding to 30.3% (1) to 46.1% (5) of odontogenic cysts. Nevertheless, although another survey also observed that the dentigerous cyst was the most common cystic lesion in children and adolescents, there was greater prevalence in the male gender (62%) (3).

Inflammatory periapical lesions corresponded to 4.5% of all lesions, with periapical granulomas representing the most common lesion. Lawoyin (2) classified the periapical granuloma in the category of inflammatory lesions and observed that this was the disease most frequently observed in this group, similar to the series of Jones and Franklin (1) who identified the periapical granuloma as the most prevalent dental pathology. Although they are common lesions, in the present study, inflammatory periapical lesions represented only 4.5% of the total number of lesions. This low prevalence in an oral pathology laboratory indicates a major concern, because it may indicate that several of these diseases are not sent for histopathological analysis.

The odontogenic tumors represented 4.12% of all lesions, with the odontoma and adenomatoid odontogenic tumor (AOT) being the most common tumors. Although other series also found odontoma and AOT as the most common odontogenic tumors in this age group, the odontoma presented a higher prevalence (1,19). On the other hand, Ajayi et al. (20) observed that ameloblastomas are the most frequent odontogenic tumors in patients younger than 19 years old in Nigeria, followed by the AOT.

In order to define the most adequate clinical approach, it is essential to establish consistent differential diagnoses. For this, the dentist should recognize the clinical features of the different lesions that may occur in children and adolescents. To the best of our knowledge, there are not previous studies that evaluated the correlation between clinical diagnosis and histopathological diagnosis. In the present series, 38.5% of the cases showed no correlation between clinical hypothesis and histopathological diagnosis.

Oral lesions series in children and adolescents are scarce in the literature, and knowledge of these data may help pediatric dentists in making precise diagnosis of oral diseases that affect this population. To establish coherent differential diagnosis is essential to guide the most adequate approach. In addition, professionals should pay special attention to the clinical and/or radiographic information of lesions sent for histopathological exam, since they are essential for the establishment of the definitive diagnosis.
